# Supplementary material for: ATF4 selectively regulates heat nociception and contributes to kinesin-mediated TRPM3 trafficking
Source: Nat Commun. 2021 Mar 3;12:1401. doi: 10.1038/s41467-021-21731-1 (PMC7930092; doi:10.1038/s41467-021-21731-1)

ATF4 selectively regulates heat nociception and contributes to kinesin-mediated TRPM3 trafficking

Xie et al.

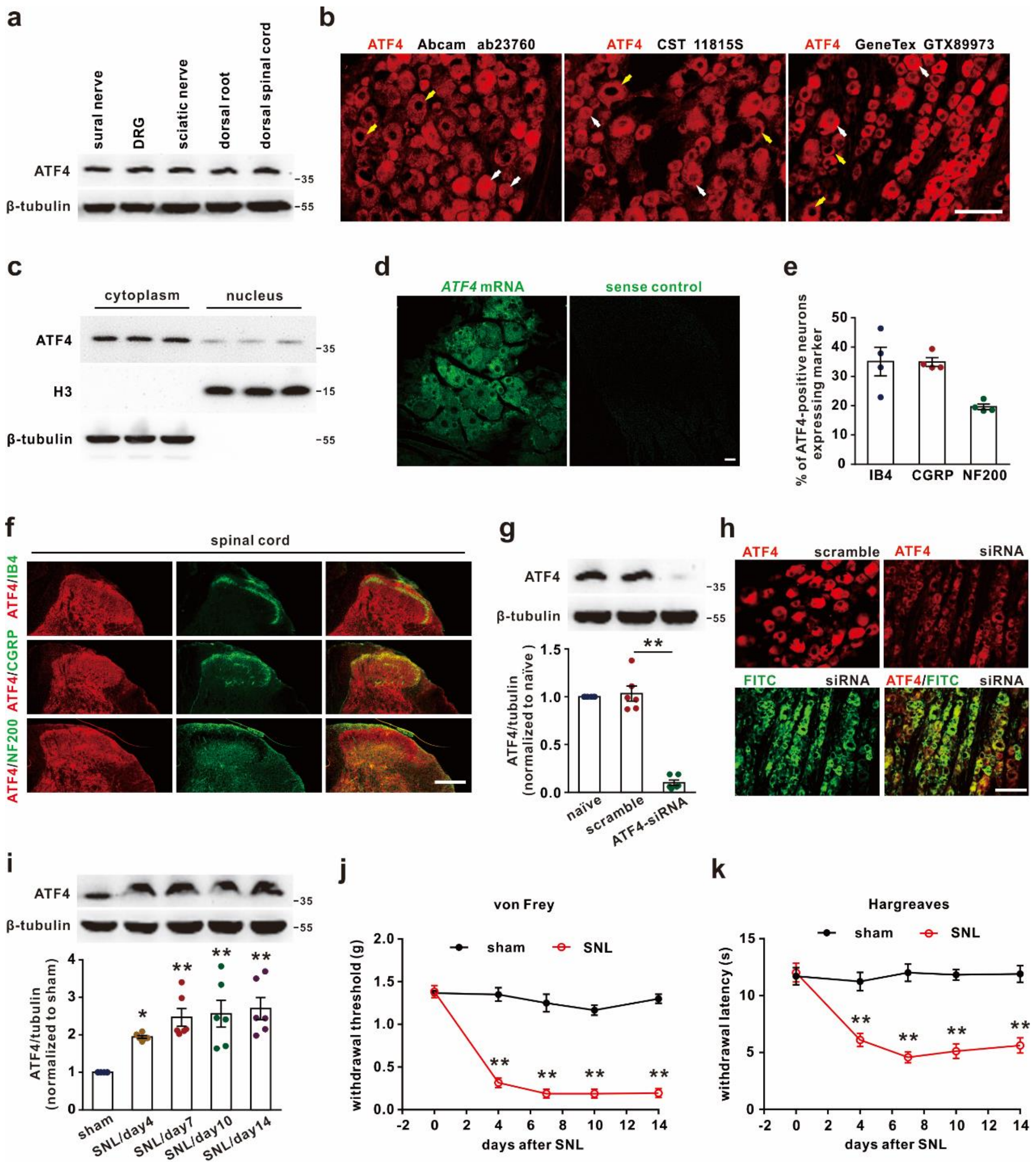

Supplementary Fig. 1 SNL increases the expression of ATF4 in DRG tissues. (**a**) ATF4 expression profile in mouse peripheral sensory nervous system. (**b**) Immunostaining for ATF4 in sensory neurons with three antibodies from different companies (Abcam, catalogue no.: ab23760; Cell Signaling Technology, catalogue no.: 11815S and GeneTex, catalogue no.: GTX89973). ATF4 was expressed in the cytoplasm of all stained neurons and distributed in the nuclei of most stained DRG neurons (white arrow); however, a small number of neurons did not express ATF4 in

the nucleus (yellow arrow). Scale bar, 100 μm. (**c**) The expression of ATF4 in cytoplasm and nuclei of mouse DRG neurons. n = 3 mice per group. (**d**) *In situ* hybridization images showing *ATF4* mRNA expression in the DRG. Scale bar, 20 μm. (**e**) The percentage of ATF4-positive DRG neurons expressing IB4, CGRP and NF200. n =4 mice per group. (**f**) Colocalization of ATF4 with cell markers (IB4, CGRP and NF200) in the dorsal spinal cord. Scale bar, 200 μm. (**g**) Expression of ATF4 in DRG tissues after ATF4 siRNA treatment. n = 6 mice per group. $F_{(2, 15)} = 122.0$, $P < 0.0001$. $^{**}P < 0.01$. (**h**) Double immunostaining for FITC-labelled ATF4 siRNA and ATF4 showing the expression of ATF4 protein in DRG neurons after ATF4 siRNA treatment. Scale bar, 100 μm. (**i**) Expression of ATF4 in the mouse DRG after SNL. n = 6 mice per group. $F_{(4, 25)} = 8.932$, $P = 0.0329$ in day 4, $P = 0.0006$ in day 7, $P = 0.0003$ in day 10, $P < 0.0001$ in day 14. $^{*}P < 0.05$, $^{**}P < 0.01$ versus the sham group. (**j, k**) SNL-induced mechanical (**j**) and thermal (**k**) hypersensitivity. n = 12 mice per group. $F_{(1, 22)} = 310.9$, $P < 0.0001$ in day 4, 7, 10 and 14 in **j**. $F_{(1, 22)} = 180.9$, $P < 0.0001$ in day 4, 7, 10 and 14 in **k**. $^{**}P < 0.01$ versus the sham group. **g**, **i,** One-way ANOVA followed by Tukey's multiple comparisons test. **j**, **k,** Two-way ANOVA followed by Bonferroni's multiple comparisons test. The error bars indicate the SEMs.

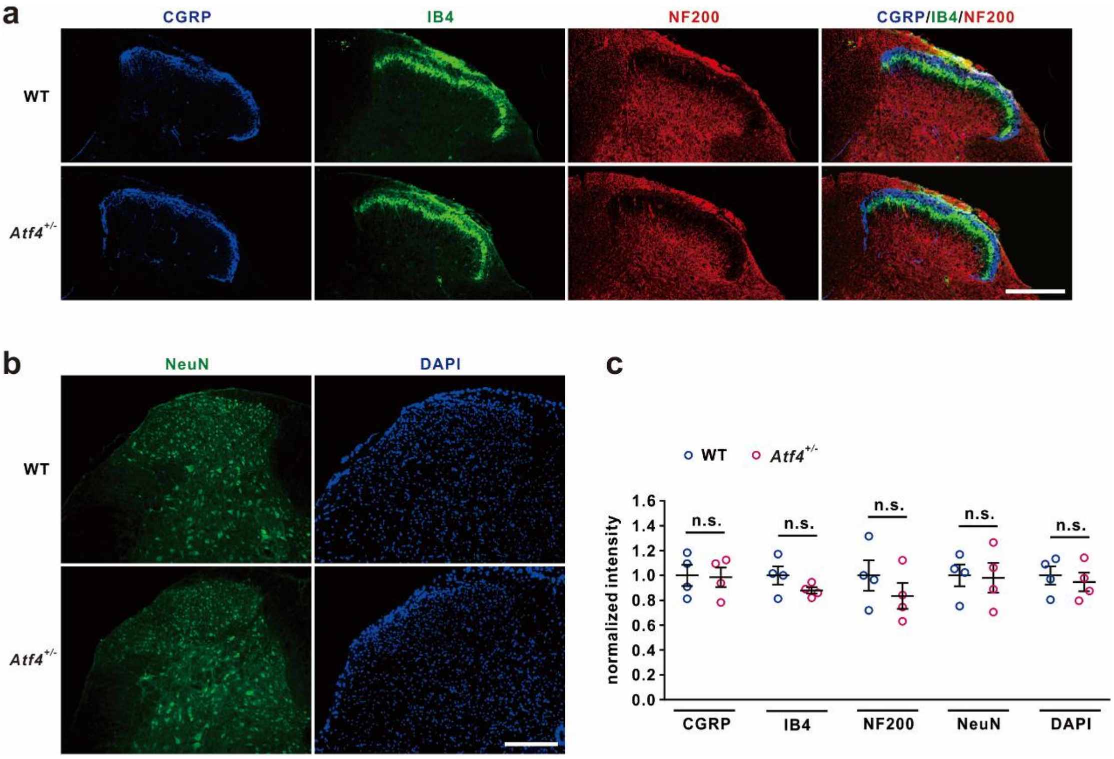

Supplementary Fig. 2 ***Atf4***$^{+/-}$ **mice display normal central innervations in the spinal dorsal horn.** (**a**) Immunostaining for CGRP, IB4 and NF200 in L4 spinal cord sections from WT and *Atf4*$^{+/-}$ mice. Scale bar, 200 µm. (**b**) NeuN immunostaining and DAPI staining of L4 spinal cord sections from WT and *Atf4*$^{+/-}$ mice. Scale bar, 200 µm. Note that heterozygous ATF4 knockout did not cause cell loss in the spinal cord. (**c**) Quantification of immunofluorescence of CGRP, IB4, NF200, NeuN and DAPI staining in the dorsal horn in WT and heterozygous mice. n = 4 mice per group. Two-tailed Independent Student's *t* test. n.s. means not significant. The error bars indicate the SEMs.

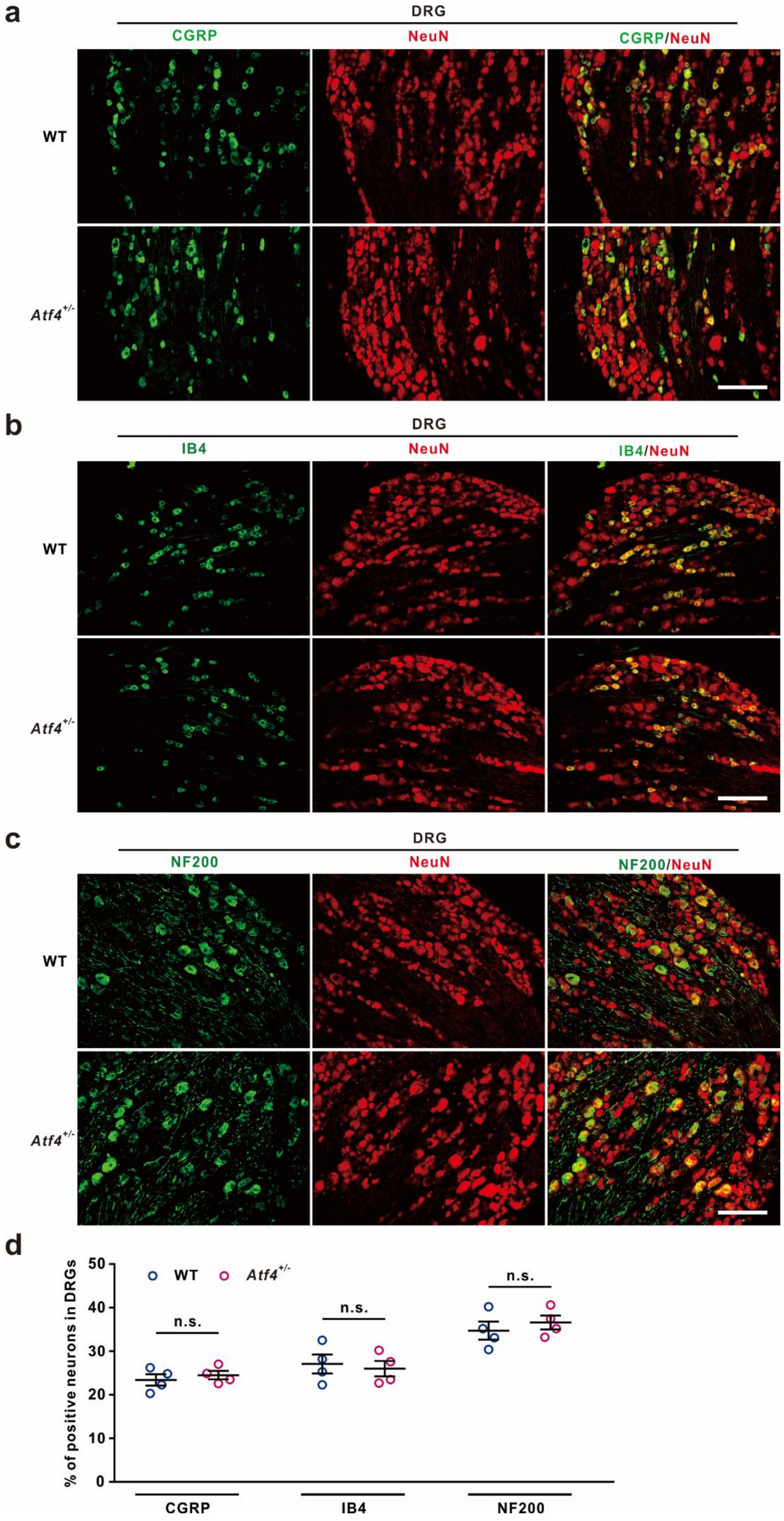

a
DRG
CGRP
NeuN
CGRP/NeuN
WT
Atf4+/-
b
DRG
IB4
NeuN
IB4/NeuN
WT
Atf4+/-
c
DRG
NF200
NeuN
NF200/NeuN
WT
Atf4+/-
d
% of positive neurons in DRGs
WT
Atf4+/-
n.s.
n.s.
n.s.
0
10
20
30
40
50
CGRP
IB4
NF200

Supplementary Fig. 3 The distribution patterns of C-fibre and A-fibre neurons are normal, and no neurons are lost in the DRG of *Atf4*$^{+/-}$ mice. (**a-c**) Immunostaining for CGRP, IB4 and NF200 in L4 DRG sections from WT and *Atf4*$^{+/-}$ mice. Scale bar, 200 μm. Note that *Atf4*$^{+/-}$ mice exhibited normal distribution patterns of different populations of sensory DRG neurons. (**d**) Quantification of the percentages of CGRP-positive, IB4-positive and NF200-positive neurons in WT and *Atf4*$^{+/-}$ mice. n = 4 mice per group. Two-tailed Independent Student's *t* test. n.s. means not significant. The error bars indicate the SEMs.

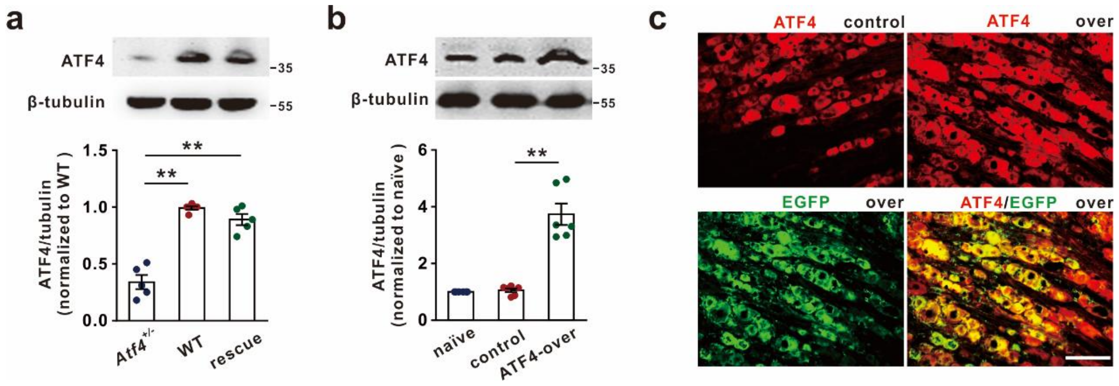

Supplementary Fig. 4 AAV-mediated overexpression or rescue of ATF4 in DRG tissues. (**a**) rAAV-CMV-*Atf4*-2A-EGFP-WPRE-PA was intrathecally injected into *Atf4*$^{+/-}$ mice to rescue the expression of ATF4 in the DRG. n = 5 mice per group. $F_{(2, 12)}$ = 56.91, P < 0.0001 in *Atf4*$^{+/-}$ vs. WT and *Atf4*$^{+/-}$ vs. rescue. (**b, c**) ATF4 expression in DRG tissues was observed 21 days after rAAV-CMV-*Atf4*-2A-EGFP-WPRE-PA intrathecally injected. n = 6 mice per group. $F_{(2, 15)}$ = 50.10, P < 0.0001. Scale bar, 100 µm. **P < 0.01. One-way ANOVA followed by Tukey's multiple comparisons test. The error bars indicate the SEMs.

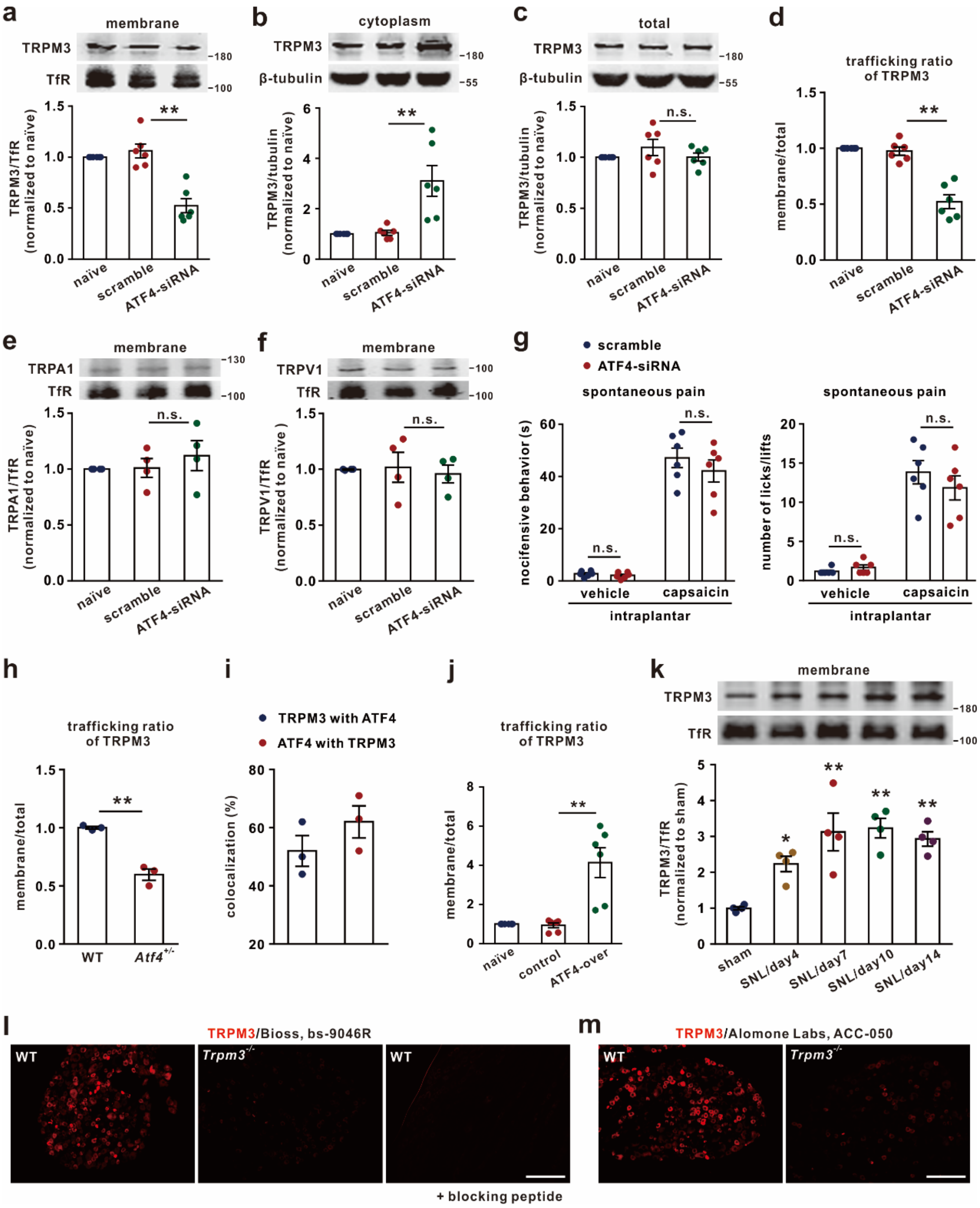

Supplementary Fig. 5 The effects of ATF4 on the membrane trafficking of TRPM3.

(**a**-**c**) TRPM3 expression in DRG membrane (**a**), cytoplasmic (**b**) and total lysate (**c**) after ATF4 siRNA treatment. n = 6 mice per group. $F_{(2, 15)} = 27.56$, $P < 0.0001$ in **a**. $F_{(2, 15)} = 11.45$, $P = 0.0026$ in **b**. $^{**}P < 0.01$, n.s. means not significant. (**d**) Changes in the TRPM3 trafficking ratio in DRG after ATF4 siRNA treatment. n = 6, $F_{(2, 15)} = 41.81$, $P < 0.0001$. $^{**}P < 0.01$. (**e, f**) TRPA1 (**e**) and TRPV1 (**f**) expression in DRG membrane

fraction after ATF4 siRNA treatment. n = 4 mice per group. n.s. means not significant. (**g**) Spontaneous pain: total duration or number of nocifensive behaviours (paw licking or flinching within 2 min) in response to intraplantar injection of capsaicin (1 nmol/paw) into the ATF4 siRNA and scrambled mice. n = 6 mice per group. n.s. means not significant. (**h**) The TRPM3 trafficking ratio in DRGs of WT and *Atf4*$^{+/-}$ mice. n = 3. t_4 = 7.996, P = 0.0013. **$P < 0.01$. (**i**) Quantification data show the colocalization rates of ATF4 with TRPM3 (colocalized yellow spots / total ATF4 positive spots) and those of TRPM3 with ATF4 (colocalized yellow spots / total TRPM3 positive spots) in DRG neurons of Fig. 5f. n = 3 mice. (**j**) Changes of TRPM3 trafficking ratio in DRG after ATF4 overexpression. n = 6. $F_{(2, 15)}$ = 16.92, P = 0.0004. **$P < 0.01$. (**k**) Membrane expression of TRPM3 in DRG tissues after SNL treatment. n = 4 mice per group. $F_{(4, 15)}$ = 9.84, P = 0.0388 in day 4, P = 0.0005 in day 7, P = 0.0003 in day 10, P = 0.0013 in day 14. *$P < 0.05$, **$P < 0.01$ versus sham group. (**l, m**) The specificity of TRPM3 antibodies (from two companies) was determined in TRPM3 knockout mice and with TRPM3 blocking peptide. Scale bar, 200 μm. **a-f, j, k,** One-way ANOVA followed by Tukey's multiple comparisons test. **g, h,** Two-tailed Independent Student's *t* test. The error bars indicate the SEMs.

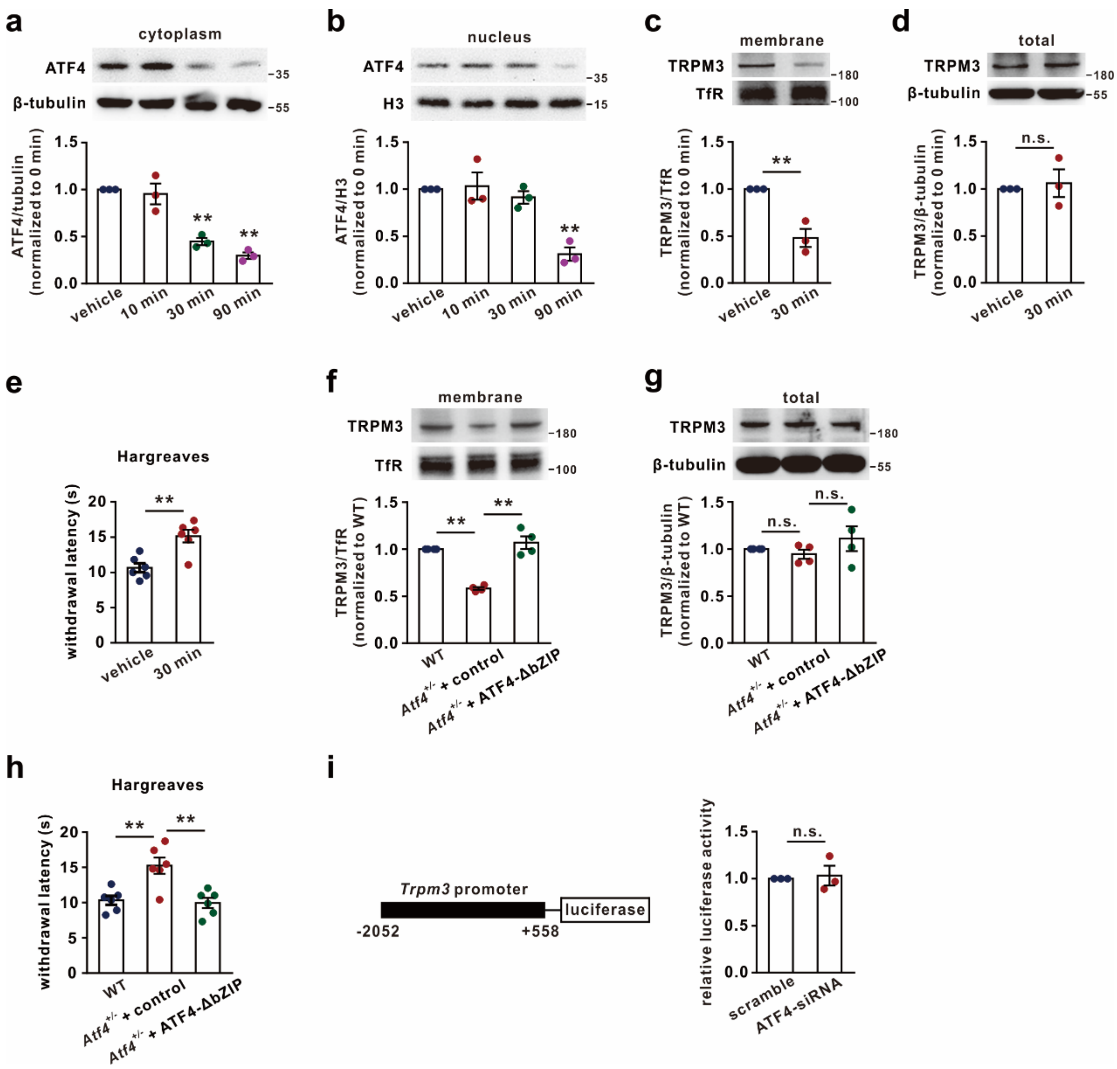

Supplementary Fig. 6 ATF4 has no transcriptional regulatory effect on TRPM3. (**a, b**) ISRIB (300 ng) was intrathecally injected, and ATF4 levels in the cytoplasm (**a**) and nuclei (**b**) of DRG neurons were measured at different time points. n = 3 mice per group. $F_{(3, 8)}$ = 34.16, P = 0.0009 in 30 min, P = 0.0002 in 90 min in **a**. $F_{(3, 8)}$ = 15.39, P = 0.0022 in 90 min in **b**. **P < 0.01 versus vehicle. (**c, d**) The effects of intrathecal injection of ISRIB on the membrane (**c**) and total (**d**) expression of TRPM3 after 30 min. n = 3 mice per group. t_4 = 5.392, P = 0.0057. **P < 0.01, n.s. means not significant. (**e**) The effects of intrathecal injection of ISRIB on behaviour in the Hargreaves test after 30 min. n = 6 mice per group. t_{10} = 4.068, P = 0.0023. **P < 0.01. (**f, g**) The effects

of overexpression of a transcriptionally inactive form of ATF4 (rAAV-CMV-*Atf4*-ΔbZIP) on the membrane (**f**) and total (**g**) expression of TRPM3 in sensory neurons of *Atf4*$^{+/-}$ mice. n = 4 mice per group. $F_{(2, 9)}$ = 43.98, P = 0.0001 in WT vs. *Atf4*$^{+/-}$ + control, P < 0.0001 in *Atf4*$^{+/-}$ + control vs. *Atf4*$^{+/-}$ + ATF4-ΔbZIP. **P < 0.01, n.s. means not significant. (**h**) The effects of overexpression of a transcriptionally inactive form of ATF4 (rAAV-CMV-*Atf4*-ΔbZIP) on behaviour in the Hargreaves test of *Atf4*$^{+/-}$ mice. n = 6 mice per group. $F_{(2, 15)}$ = 11.4, P = 0.0033 in WT vs. *Atf4*$^{+/-}$ + control, P = 0.0018 in *Atf4*$^{+/-}$ + control vs. *Atf4*$^{+/-}$ + ATF4-ΔbZIP. **P < 0.01. (**i**) Knockdown of ATF4 did not alter the luciferase activity of the *Trpm3* promoter. This experiment was repeated three times, n.s. means not significant. **a, b, f, g, h,** One-way ANOVA followed by Tukey's multiple comparisons test. **c-e, i,** Two-tailed Independent Student's t test. The error bars indicate the SEMs.

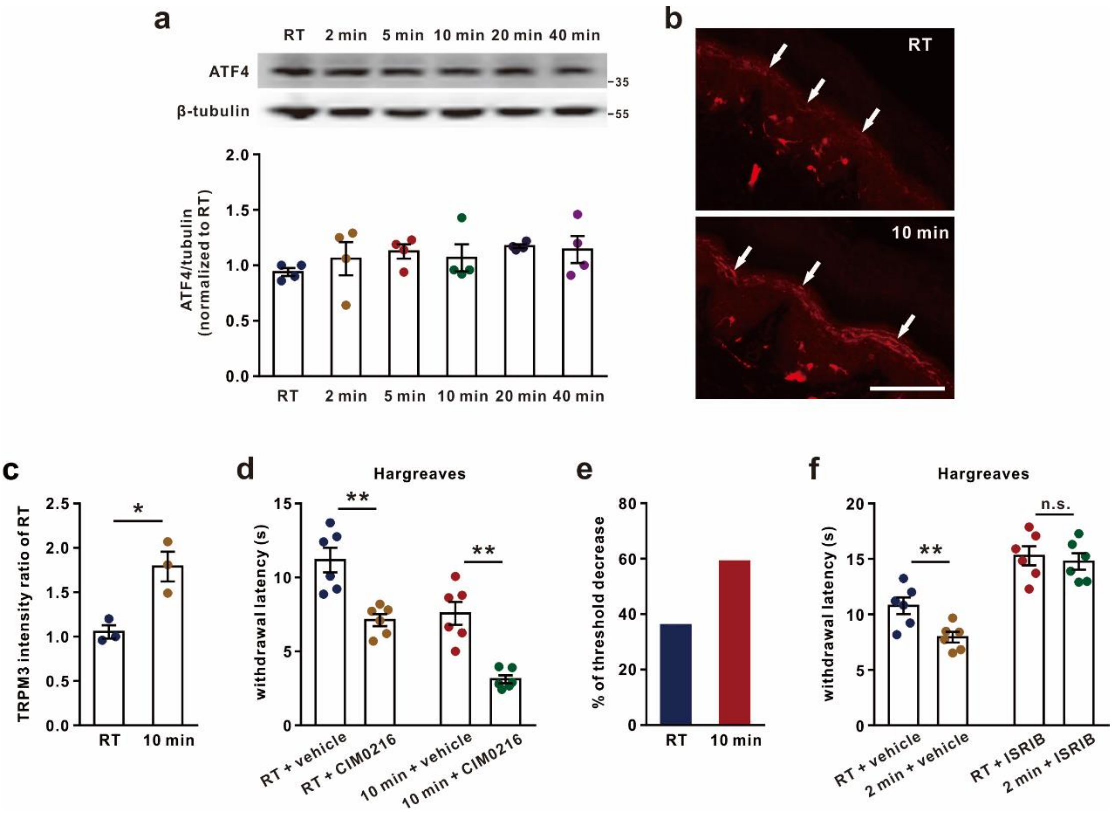

Supplementary Fig. 7 Thermal stimulation increases the expression and function of TRPM3 in nerve endings. (**a**) The expression of ATF4 in mouse DRG tissues was evaluated at different time points after heat stimulation. n = 4 mice per group. (**b, c**) Immunostaining of TRPM3 in the nerve endings of RT or heat stimulation mice (**b**) and quantification of immunofluorescence of TRPM3 staining in the nerve endings of RT and heat stimulation mice (**c**). n = 3 mice per group. Three to five sections from each animal were included for quantification. $t_4 = 4.016$, $P = 0.0159$. Scale bar, 100 µm. (**d**) The change of withdrawal latency after TRPM3 agonist (CIM0216, 2.5 nmol/paw) injected into the hindpaw of RT and heat stimulation mice. n = 6 mice per group. $t_{10} = 4.391$, $P = 0.0014$ in RT + vehicle vs. RT + CIM0216. $t_{10} = 5.424$, $P = 0.0003$ in 10 min + vehicle vs. 10 min + CIM0216. (**e**) The percentage of thermal withdrawal threshold decreases [(vehicle - CIM0216)/vehicle] in heat stimulation and RT mice after CIM0216 treatment. n = 6 mice per group. (**f**) The effect of intrathecal injection of ISRIB for 30 minutes on the decrease of heat threshold in mice after paws heat

stimulation at 2 min. n = 6 mice per group. $t_{10} = 3.184$, $P = 0.0097$ in RT + vehicle vs. 2 min + vehicle. *$P < 0.05$, **$P < 0.01$, n.s. means not significant. **a**, One-way ANOVA followed by Tukey's multiple comparisons test. **c, d, f,** Two-tailed Independent Student's *t* test. The error bars indicate the SEMs.

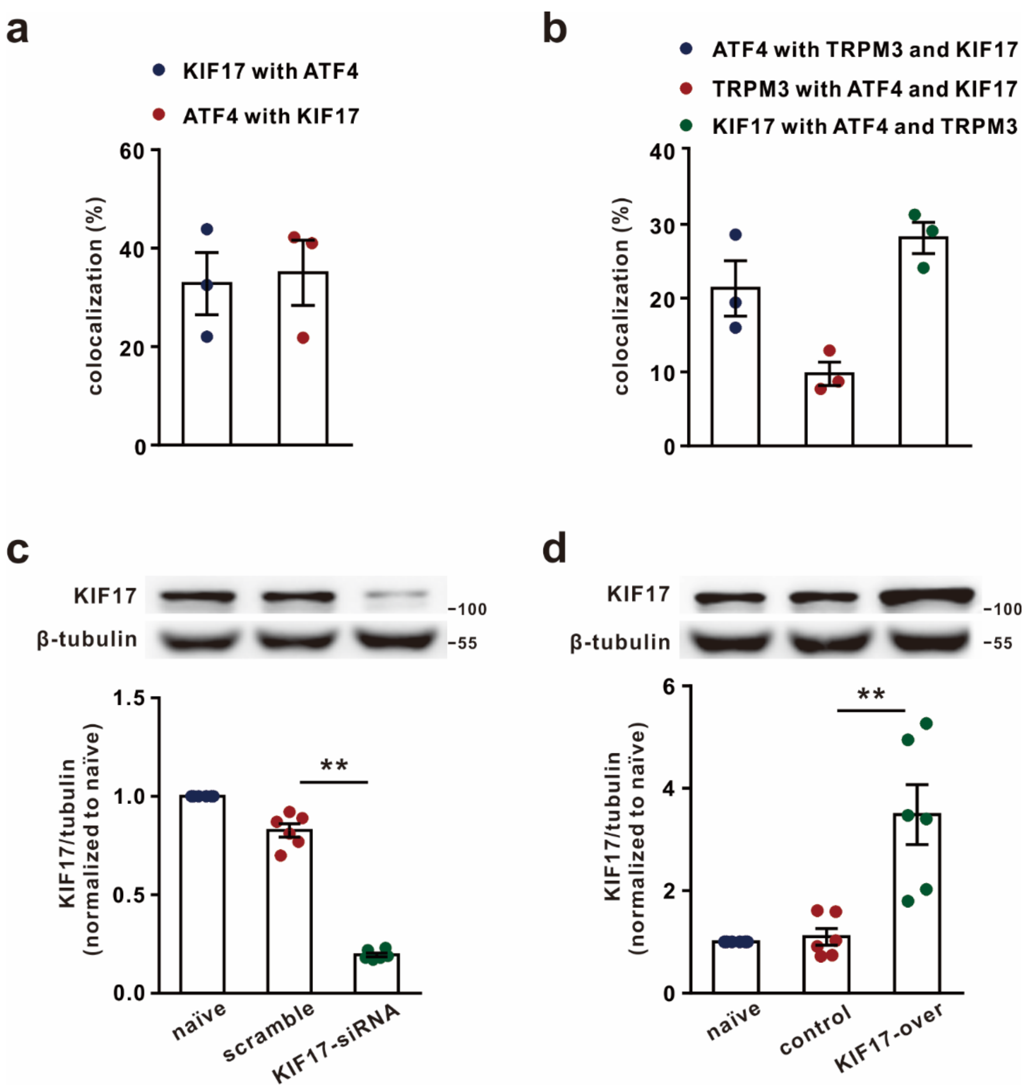

Supplementary Fig. 8 Knockdown or overexpression of KIF17 in DRG tissues. (**a**) Quantification data show colocalization rates of ATF4 with KIF17 (colocalized yellow spots / total ATF4 positive spots) and those of KIF17 with ATF4 (colocalized yellow spots / total KIF17 positive spots) in DRG neurons of Fig. 7g. n = 3 mice. (**b**) Quantification data show colocalization rates of ATF4 with TRPM3 and KIF17 (colocalized white spots / total ATF4 positive spots), of TRPM3 with ATF4 and KIF17 (colocalized white spots / total TRPM3 positive spots) and those of KIF17 with ATF4 and TRPM3 (colocalized white spots / total KIF17 positive spots) in cultured DRG neurons of Fig. 8c. n = 3 cultures. (**c**) KIF17 expression in DRG tissues after intrathecal administration of KIF17 siRNA. n = 6 mice per group. $F_{(2, 15)} = 435.7$, $P < 0.0001$. (**d**)

KIF17 expression in DRG tissues after intrathecal administration of rAAV-CMV-*Kif17*-2A-EGFP-WPRE-PA. n = 6 mice per group. $F_{(2, 15)} = 16.05$, $P = 0.0006$. **$P < 0.01$. One-way ANOVA followed by Tukey's multiple comparisons test. The error bars indicate the SEMs.

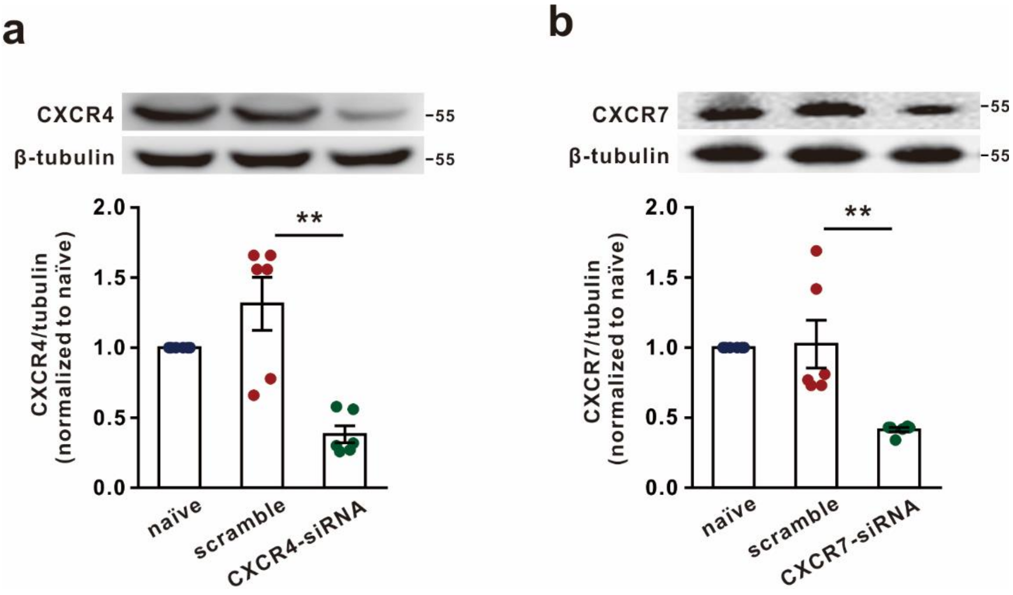

Supplementary Fig. 9 Knockdown of CXCR4 or CXCR7 expression in DRG tissues. (**a**) CXCR4 expression in DRG tissues after intrathecal administration of CXCR4 siRNA. n = 6 mice per group. $F_{(2,\ 15)} = 17.11$, $P = 0.0001$. (**b**) CXCR7 expression in DRG tissues after intrathecal administration of CXCR7 siRNA. n = 6 mice per group. $F_{(2,\ 15)} = 12.04$, $P = 0.0016$. $^{**}P < 0.01$. **a, b,** One-way ANOVA followed by Tukey's multiple comparisons test. The error bars indicate the SEMs.

Supplementary Fig. 10 Unprocessed scans of immunoblots shown in the figures.

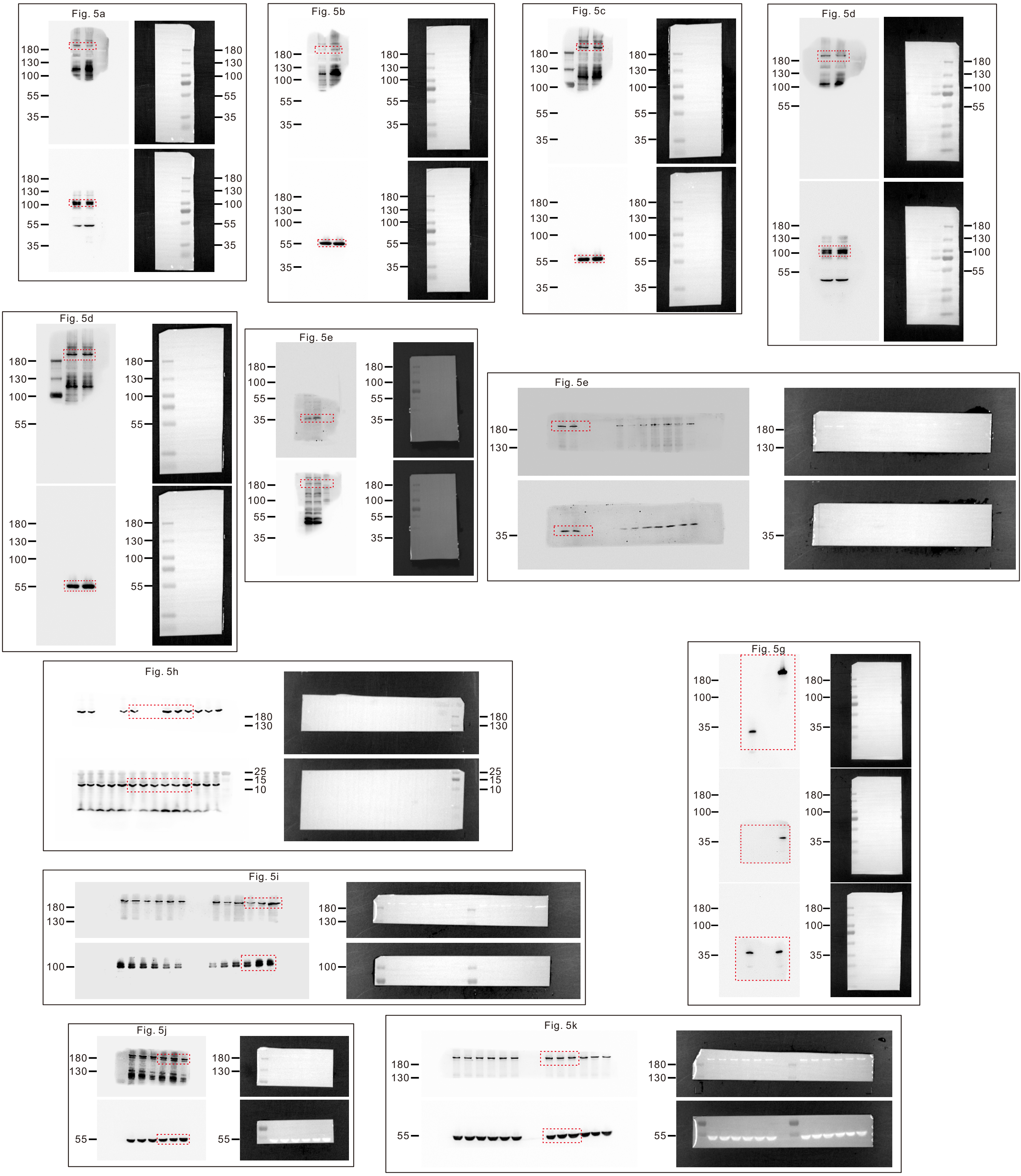

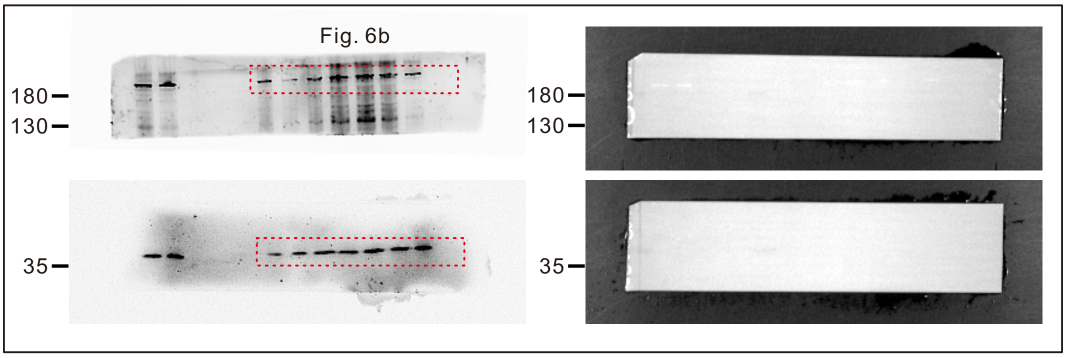
Fig. 6b
180
130
35
180
130
35

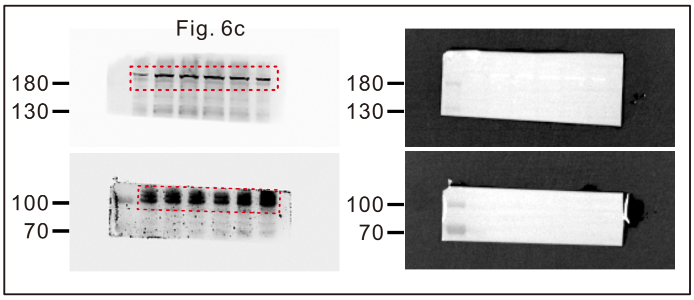
Fig. 6c
180
130
100
70
180
130
100
70

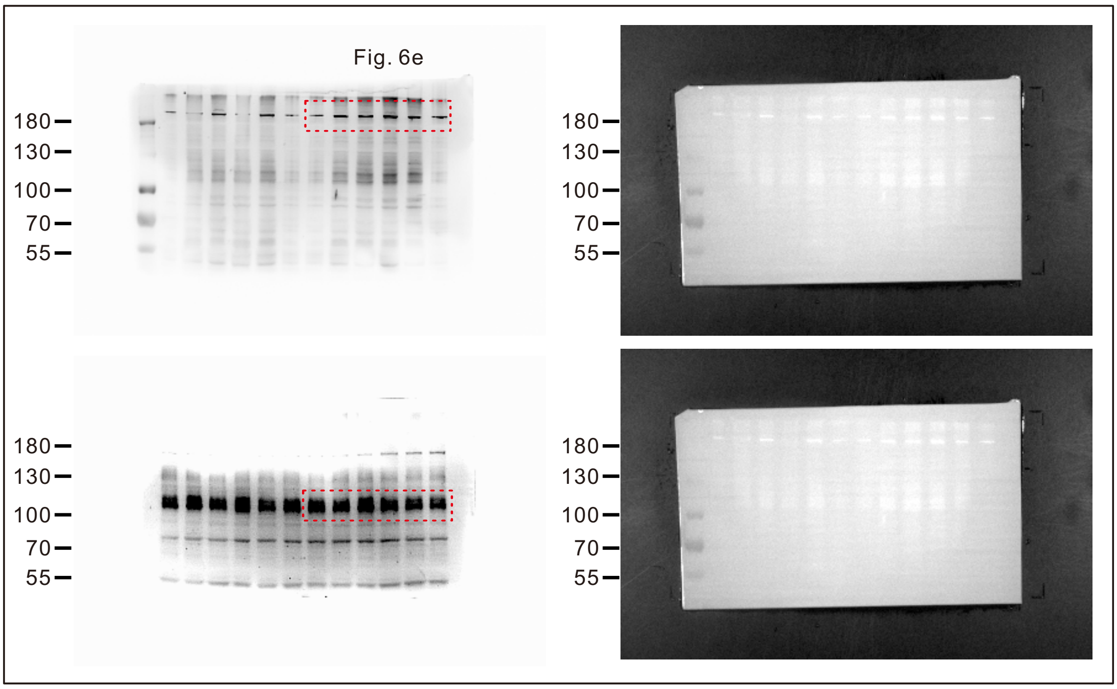
Fig. 6e
180
130
100
70
55
180
130
100
70
55
180
130
100
70
55
180
130
100
70
55

Fig. 7a

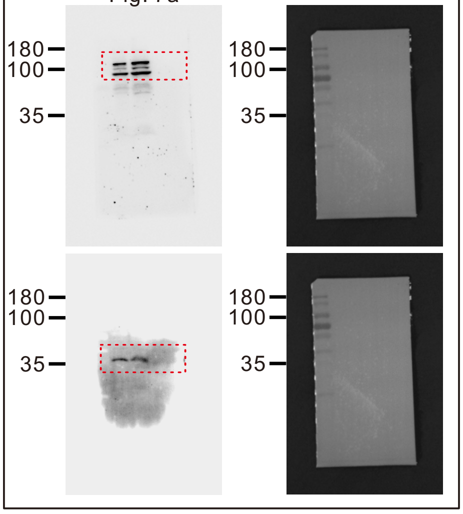

Fig. 7b

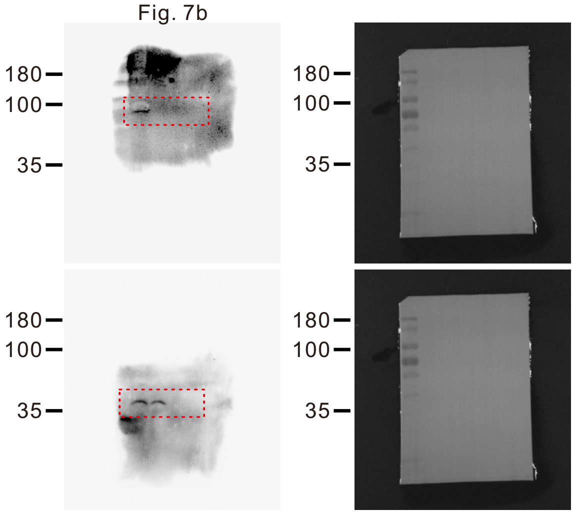

Fig. 7c

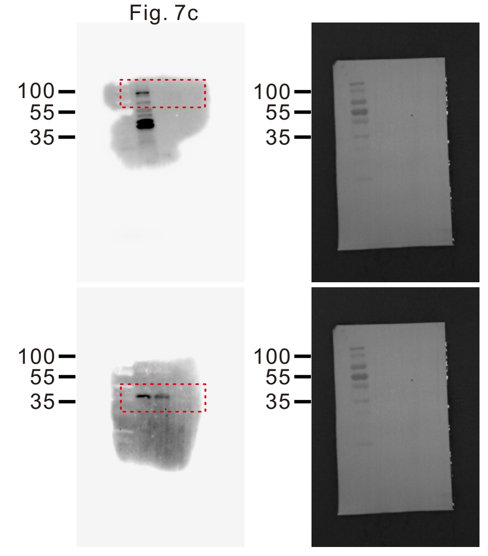

Fig. 7d

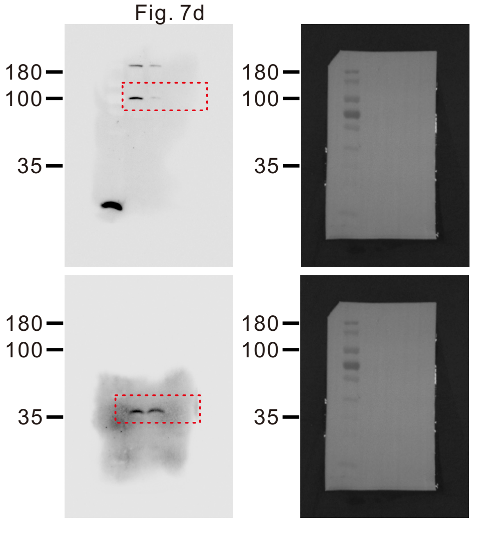

Fig. 7e

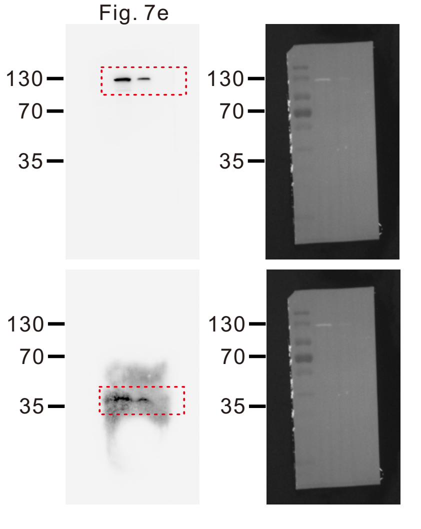

Fig. 7f

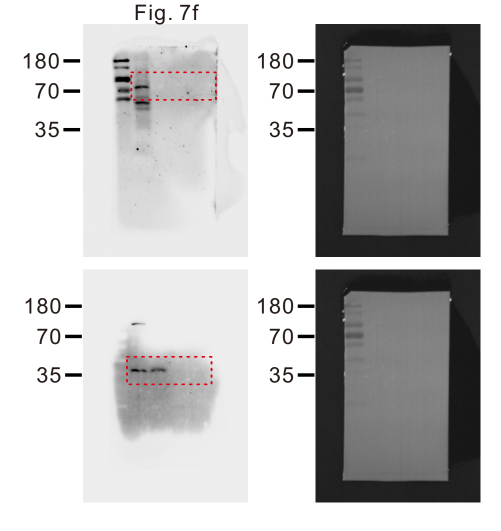

Fig. 7h

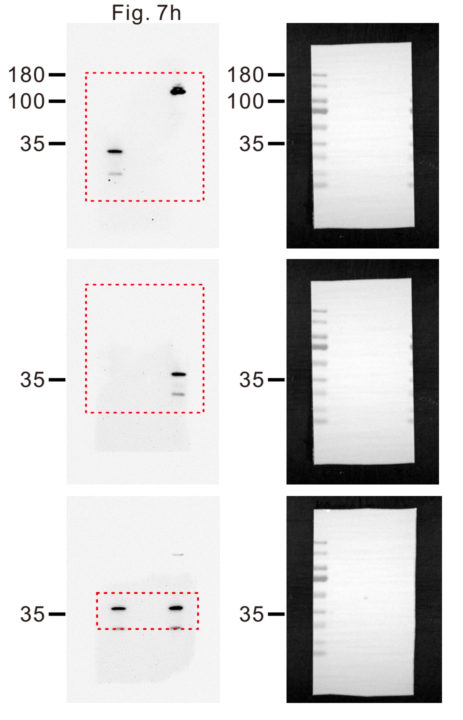

Fig. 7i

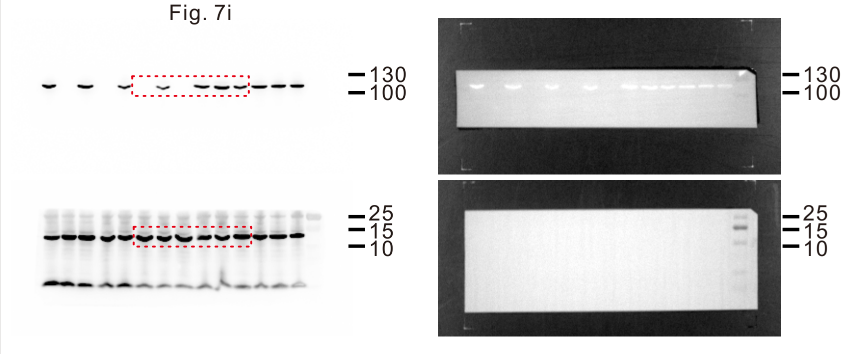

Fig. 7j

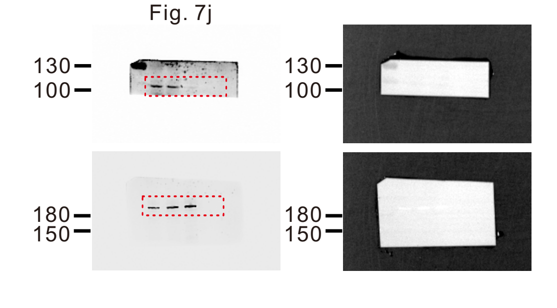

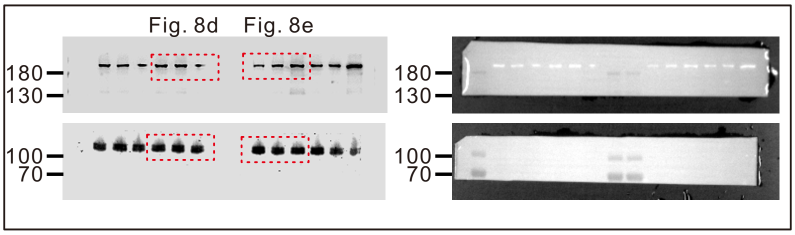
Fig. 8d
Fig. 8e
180
130
100
70
180
130
100
70

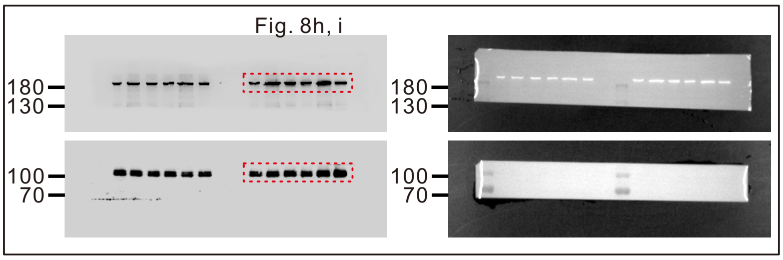
Fig. 8h, i
180
130
100
70
180
130
100
70

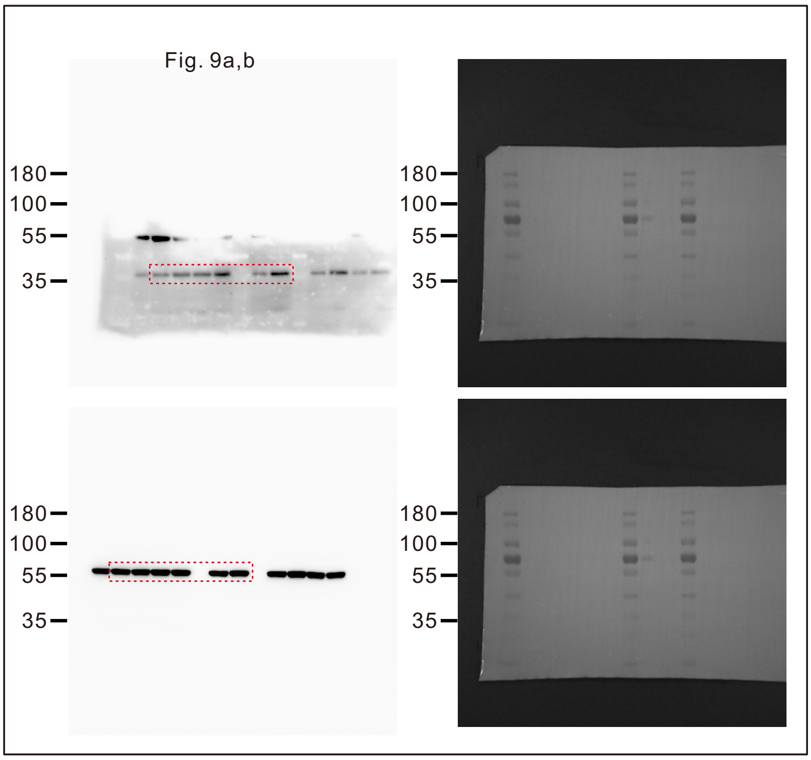
Fig. 9a,b
180
100
55
35
180
100
55
35
180
100
55
35
180
100
55
35

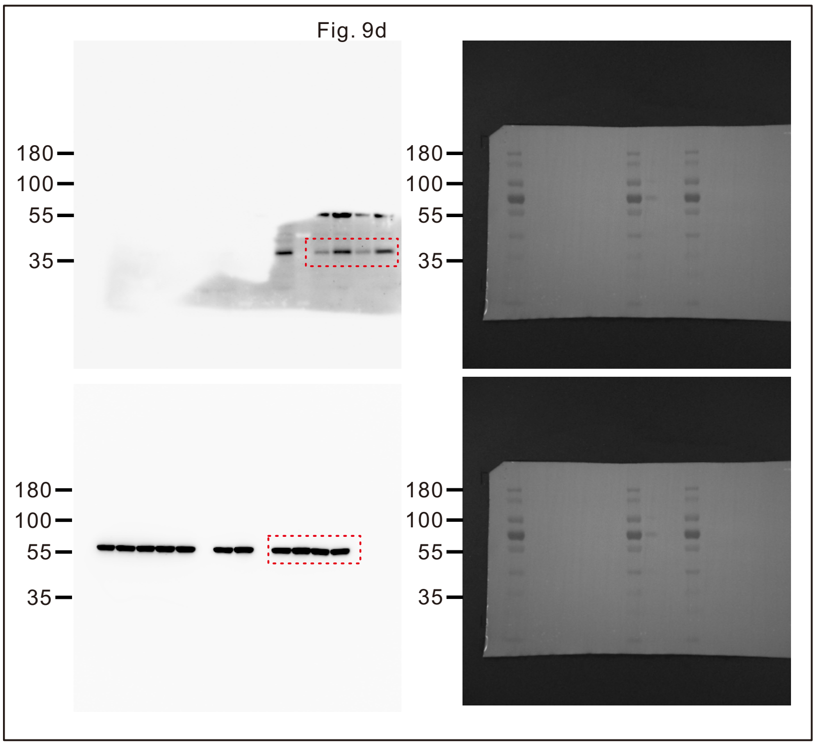
Fig. 9d
180
100
55
35
180
100
55
35
180
100
55
35
180
100
55
35

Fig. S1a, g

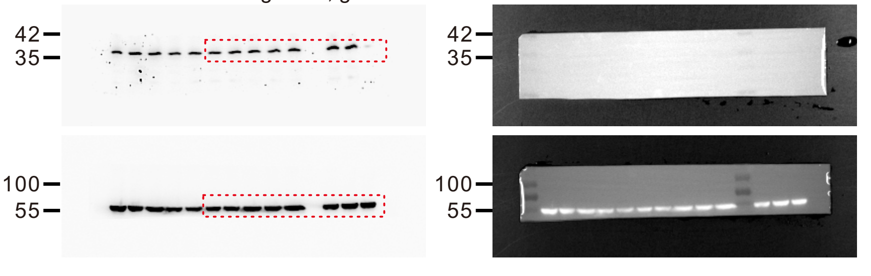

Fig. S1c

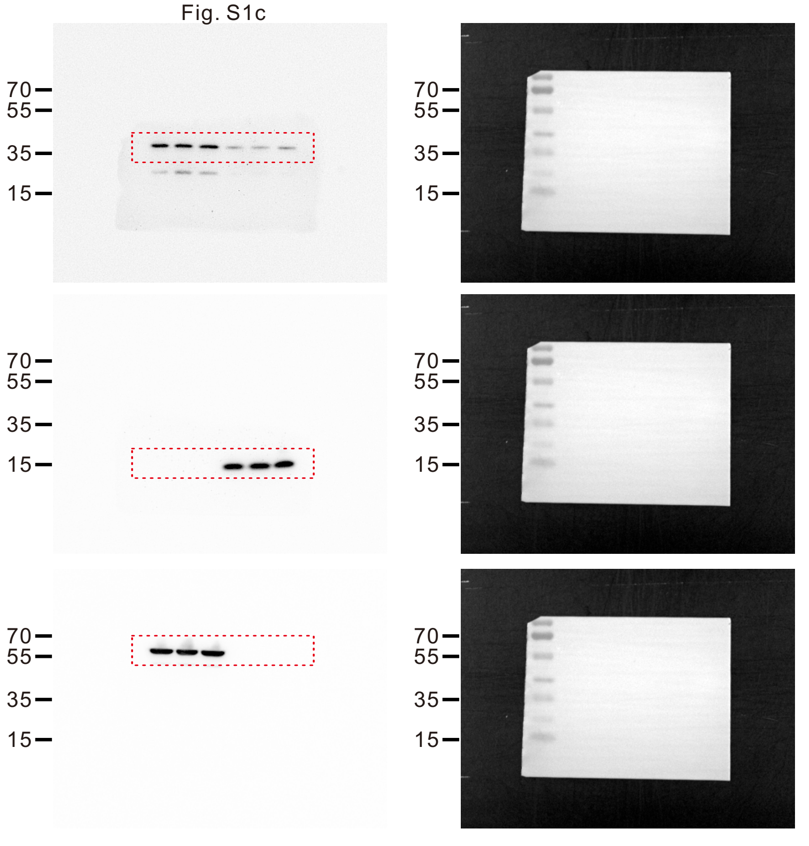

Fig. S1i

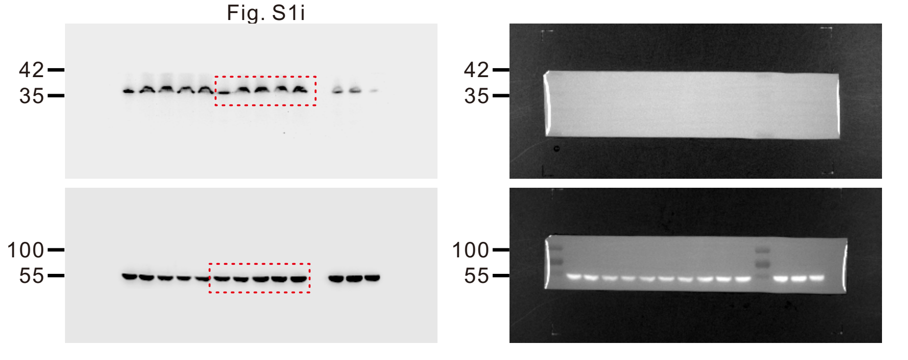

Fig. S4a

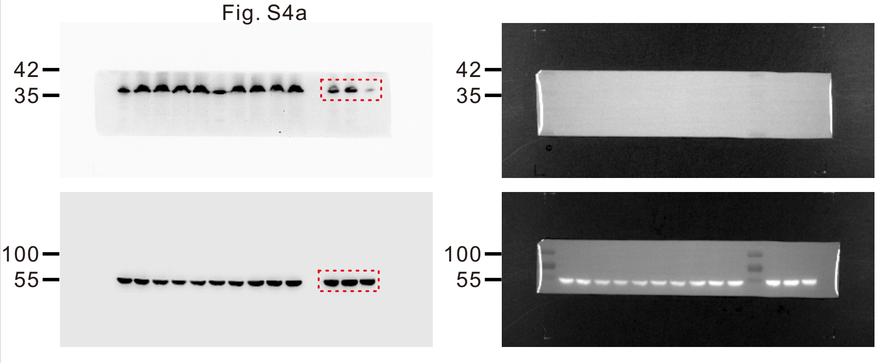

Fig. S4b

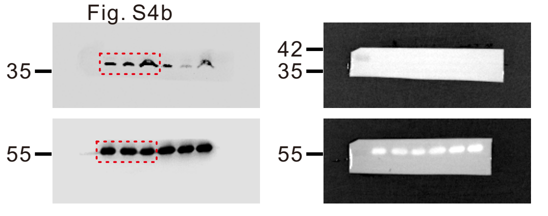

Fig. S5a

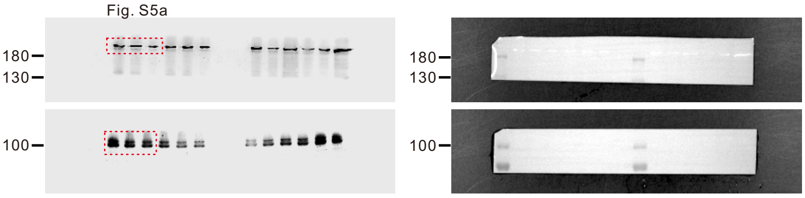

Fig. S5b

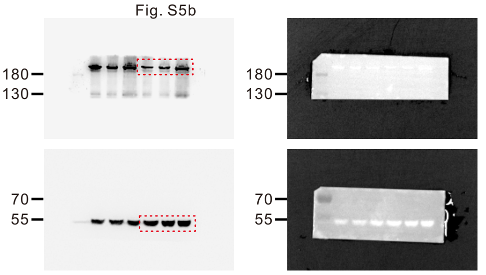

Fig. S5c

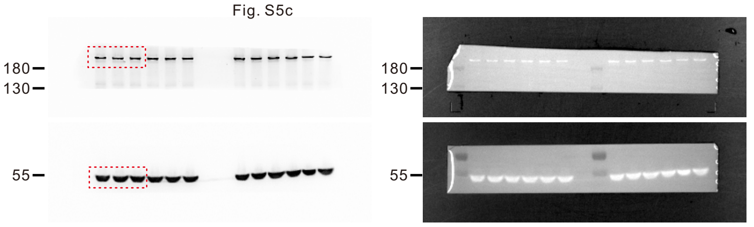

Fig. S5f

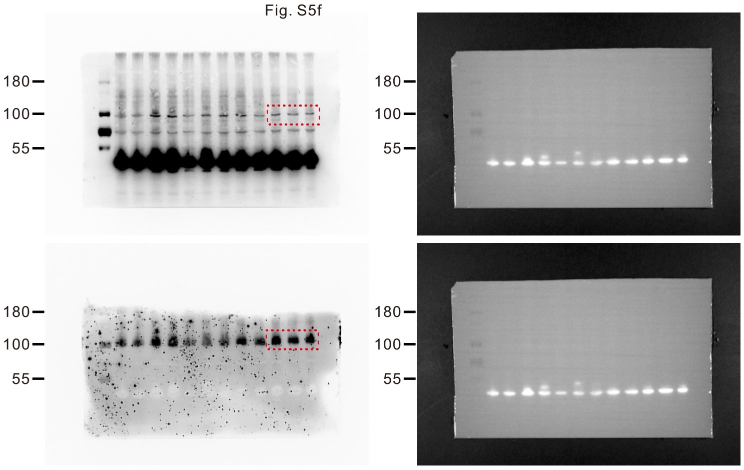

Fig. S5e

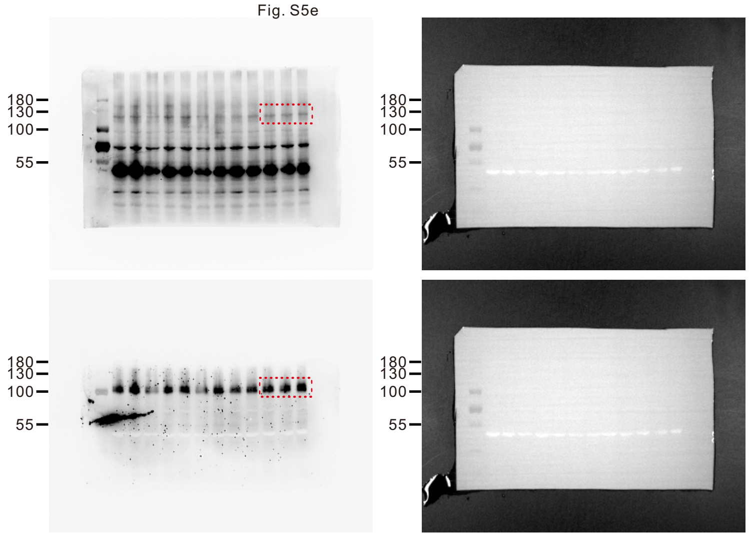

Fig. S5k

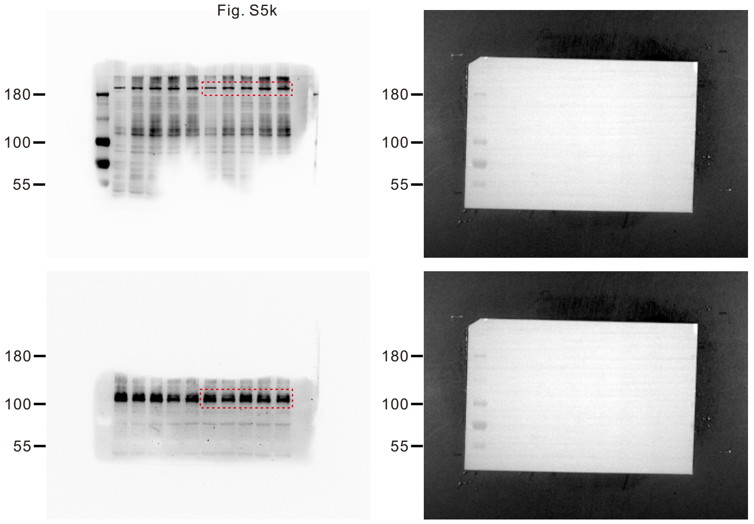

Fig. S6a

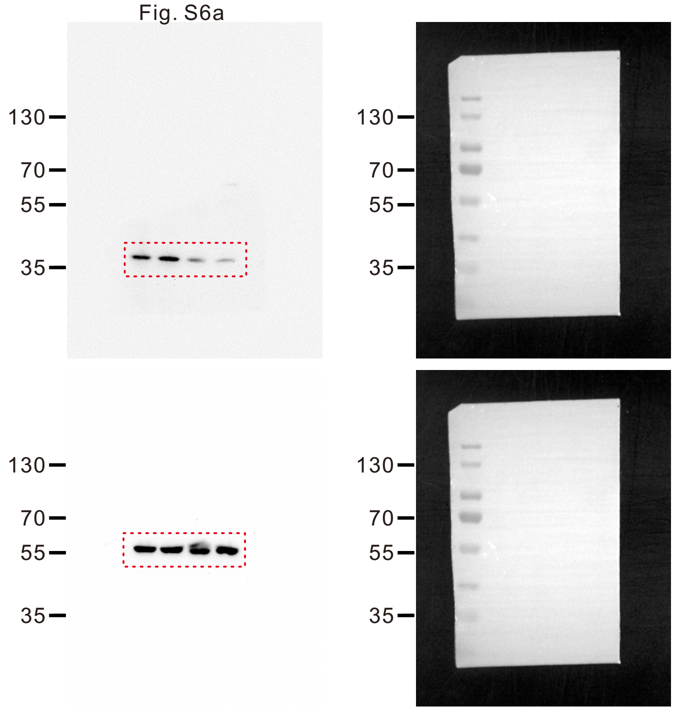

Fig. S6b

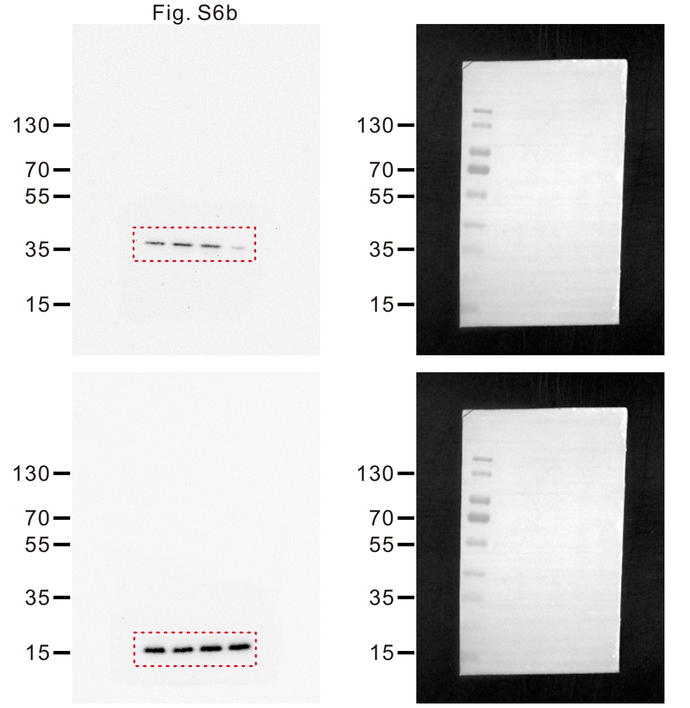

Fig. S6c

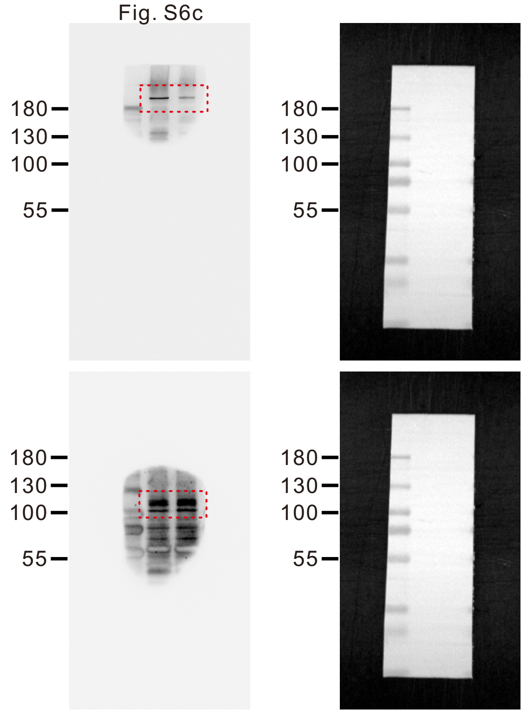

Fig. S6d

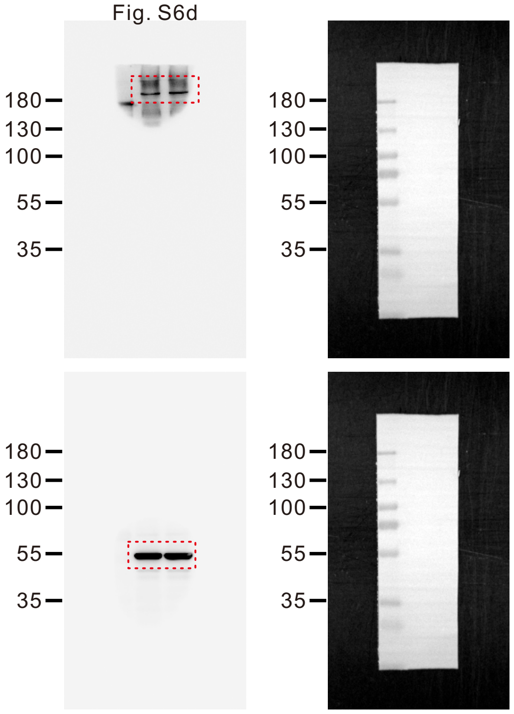

Fig. S6f

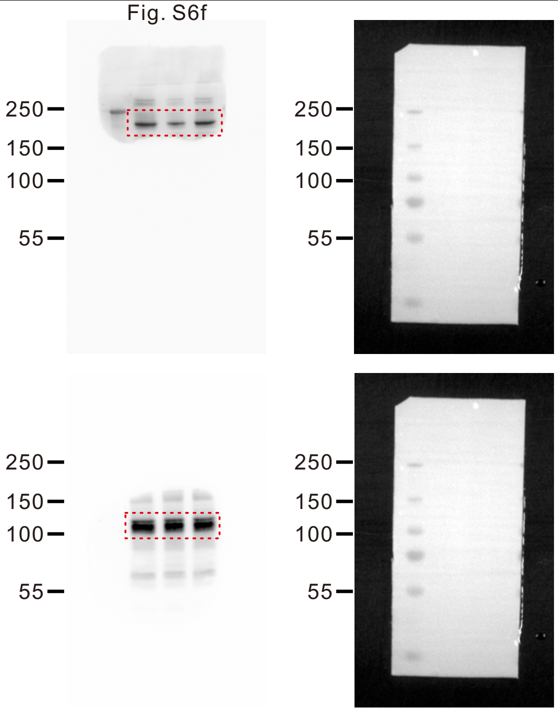

Fig. S6g

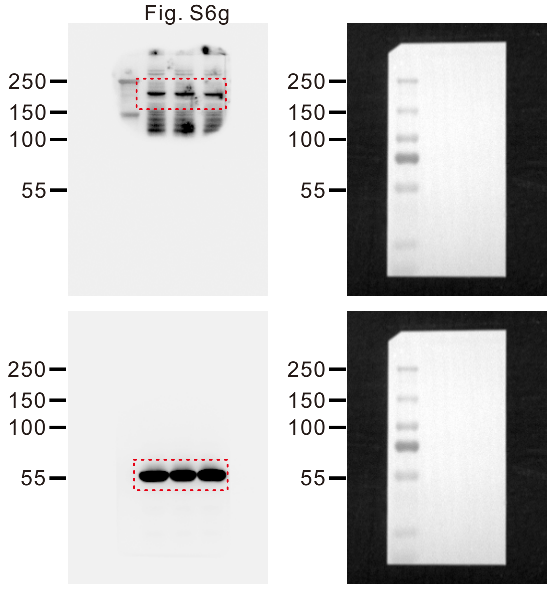

Fig. S7a

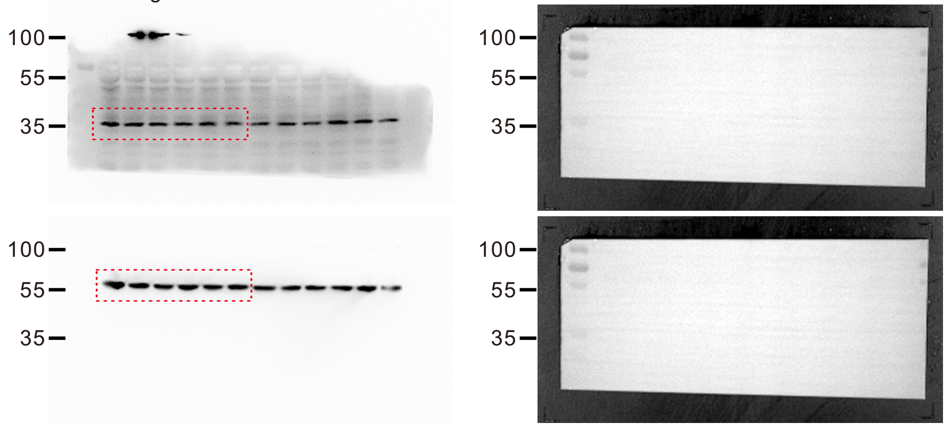

Fig. S8c,d

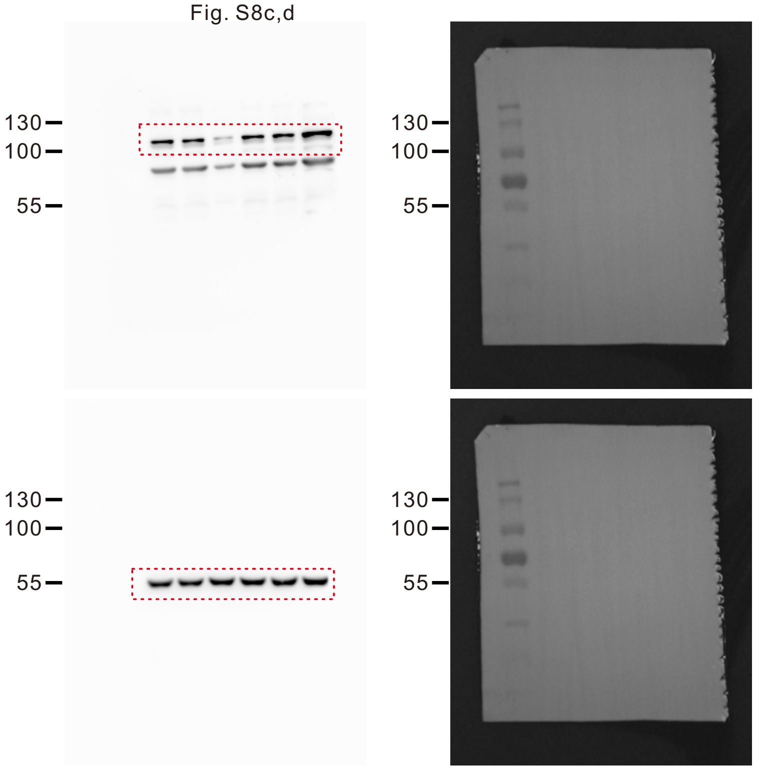

Fig. S9a

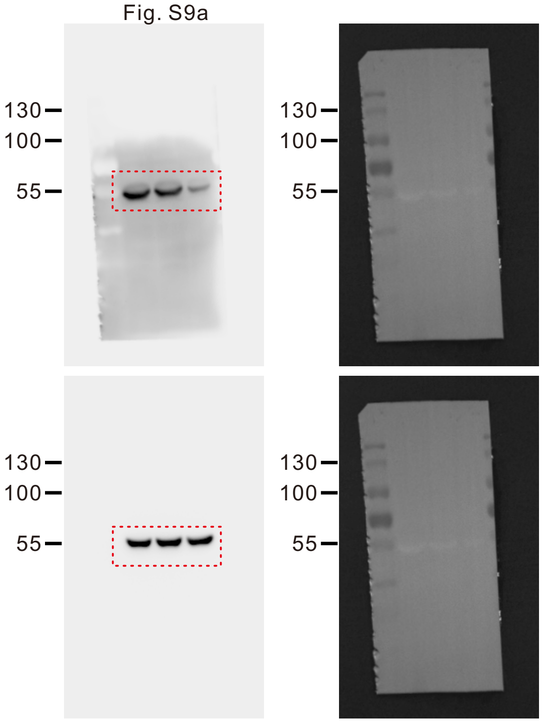

Fig. S9b

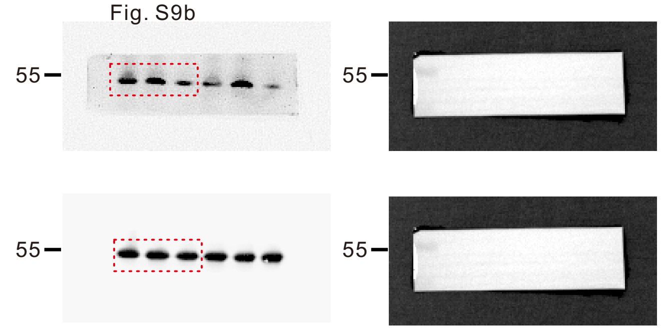

Supplement: Supplementary file 1 — Supplementary Information [file 41467_2021_21731_MOESM1_ESM.pdf]
