# Supplementary material for: ATF4 selectively regulates heat nociception and contributes to kinesin-mediated TRPM3 trafficking
Source: Nat Commun. 2021 Mar 3;12:1401. doi: 10.1038/s41467-021-21731-1 (PMC7930092; doi:10.1038/s41467-021-21731-1)
Supplement: Supplementary file 3 — Description of Additional Supplementary Files [file 41467_2021_21731_MOESM3_ESM.pdf]

Description of Additional Supplementary Files

Title: **Supplementary Movie 1**

Description: **The colocalization of KIF17 (red), ATF4 (green) and TRPM3 (blue) in cultured DRG neurons.**
