# Supplementary material for: ATF4 selectively regulates heat nociception and contributes to kinesin-mediated TRPM3 trafficking
Source: Nat Commun. 2021 Mar 3;12:1401. doi: 10.1038/s41467-021-21731-1 (PMC7930092; doi:10.1038/s41467-021-21731-1)
Supplement: Supplementary file 5 — Reporting Summary [file 41467_2021_21731_MOESM5_ESM.pdf]

nature research

Corresponding author(s): Xian-Guo Liu, Xiao-Long Zhang

Last updated by author(s): Feb 8, 2021

Reporting Summary

Nature Research wishes to improve the reproducibility of the work that we publish. This form provides structure for consistency and transparency in reporting. For further information on Nature Research policies, see our Editorial Policies and the Editorial Policy Checklist.

Statistics

For all statistical analyses, confirm that the following items are present in the figure legend, table legend, main text, or Methods section.

n/a	Confirmed	
☐	☒	The exact sample size (n) for each experimental group/condition, given as a discrete number and unit of measurement
☐	☒	A statement on whether measurements were taken from distinct samples or whether the same sample was measured repeatedly
☐	☒	The statistical test(s) used AND whether they are one- or two-sided<br>*Only common tests should be described solely by name; describe more complex techniques in the Methods section.*
☒	☐	A description of all covariates tested
☐	☒	A description of any assumptions or corrections, such as tests of normality and adjustment for multiple comparisons
☐	☒	A full description of the statistical parameters including central tendency (e.g. means) or other basic estimates (e.g. regression coefficient) AND variation (e.g. standard deviation) or associated estimates of uncertainty (e.g. confidence intervals)
☐	☒	For null hypothesis testing, the test statistic (e.g. F, t, r) with confidence intervals, effect sizes, degrees of freedom and P value noted<br>*Give P values as exact values whenever suitable.*
☒	☐	For Bayesian analysis, information on the choice of priors and Markov chain Monte Carlo settings
☒	☐	For hierarchical and complex designs, identification of the appropriate level for tests and full reporting of outcomes
☒	☐	Estimates of effect sizes (e.g. Cohen's d, Pearson's r), indicating how they were calculated

Our web collection on statistics for biologists contains articles on many of the points above.

Software and code

Policy information about availability of computer code

Data collection	Electrophysiological data were collected by PULSE software (v8.80); Western data were collected by Tanon GelCap software (2.0); Immunohistochemistry data were collected by Nikon NIS-Elements software (4.50). Plantar Test apparatus (IITC Life Science Inc.) for Hargreaves test.
Data analysis	We used Prism 7.0, , Tanon Gis (4.20) and Image-Pro Plus 6.0 to analyze the data in this study.

For manuscripts utilizing custom algorithms or software that are central to the research but not yet described in published literature, software must be made available to editors and reviewers. We strongly encourage code deposition in a community repository (e.g. GitHub). See the Nature Research guidelines for submitting code & software for further information.

Data

Policy information about availability of data

All manuscripts must include a data availability statement. This statement should provide the following information, where applicable:

- Accession codes, unique identifiers, or web links for publicly available datasets
- A list of figures that have associated raw data
- A description of any restrictions on data availability

All the figures listed in the manuscript have associated raw data and provided as a source data file. No restrictions on data availability. All data supporting this study and its findings are available from the corresponding author upon reasonable request.

Field-specific reporting

Please select the one below that is the best fit for your research. If you are not sure, read the appropriate sections before making your selection.

- [x] Life sciences
- [ ] Behavioural & social sciences
- [ ] Ecological, evolutionary & environmental sciences

For a reference copy of the document with all sections, see nature.com/documents/nr-reporting-summary-flat.pdf

Life sciences study design

All studies must disclose on these points even when the disclosure is negative.

Sample size	Sample sizes for behavioral, cellular biological, histochemical and electro-physiological studies were based on previous experiments from our laboratory and others (PMID: 32868747, PMID: 29588412, PMID: 31446225, PMID: 33433144).
Data exclusions	No data exclusions.
Replication	All attempts at replication were successful and each experiment was repeated at least three times under similar condition.
Randomization	The samples and animals were randomly assigned to experimental groups using a computer-generated table of random numbers.
Blinding	The investigators were blind to the group allocation during data collection and analysis.

Reporting for specific materials, systems and methods

We require information from authors about some types of materials, experimental systems and methods used in many studies. Here, indicate whether each material, system or method listed is relevant to your study. If you are not sure if a list item applies to your research, read the appropriate section before selecting a response.

Materials & experimental systems

n/a	Involved in the study	
☐	☒	Antibodies
☐	☒	Eukaryotic cell lines
☒	☐	Palaeontology and archaeology
☐	☒	Animals and other organisms
☒	☐	Human research participants
☒	☐	Clinical data
☒	☐	Dual use research of concern

Methods

n/a	Involved in the study	
☒	☐	ChIP-seq
☒	☐	Flow cytometry
☒	☐	MRI-based neuroimaging

Antibodies

Antibodies used	Goat anti-ATF4 (GeneTex, GTX89973), Rabbit anti-ATF4 (Abcam, ab23760), Rabbit anti-ATF4 (CST, 11815S), Rabbit anti-TRPM3 (Bioss, bs-9046R), Rabbit anti-TRPM3 (Alomone, ACC-050), Rabbit anti-TRPV1 (Alomone Labs, ACC-030), Rabbit anti-TRPA1 (ABclonal Technology, A12544), Mouse anti-TfR (Thermo Fisher Scientific, 13-6800), Goat anti-KIF3A (Santa Cruz Biotechnology, sc-18745), Rabbit anti-KIF3B (Santa Cruz Biotechnology, sc-50456), Mouse anti-KIF17 (Santa Cruz Biotechnology, sc-137040), Rabbit anti-KIF5B (Abcam, ab167429), Rabbit anti-KIF5A (Abcam, ab5628), Rabbit anti-KIFC2 (Abcam, ab3476), Mouse anti-beta Tubulin (Arigo Biolaboratories, ARG62347), Mouse anti-CGRP (Abcam, ab81887), Goat anti-CGRP (Abcam, ab36001), Isolectin B4-FITC (Sigma, L2895), Mouse anti-NF200 (Sigma, N0142), Rabbit anti-Flag (Cell Signaling Technology, 14793), Rabbit anti-His (Cell Signaling Technology, 12698), Mouse anti-NeuN (Millipore, MAB377), Rabbit anti-NeuN (Abcam, ab177487), Rabbit anti-CXCR4 (Abcam, ab124824), Rabbit anti-CXCR7 (Abcam, ab72100), Cy3 donkey anti-rabbit (IHC, Jackson, 711-165-152), Cy3 donkey anti-mouse (IHC, Jackson, 715-165-150), Cy3 donkey anti-goat (IHC, Jackson, 705-165-003), FITC donkey anti-rabbit (IHC, Jackson, 711-095-152), FITC donkey anti-mouse (IHC, Jackson, 715-095-150), FITC donkey anti-goat (IHC, Jackson, 705-095-003), Alexa 647 donkey anti-goat (IHC, Invitrogen, A32849), Alexa 405 donkey anti-rabbit (IHC, Invitrogen, A48258), Alexa 405 donkey anti-goat (IHC, Invitrogen, A48259), Peroxidase-labeled goat anti-rabbit (WB, SeraCare, 074-1506), Peroxidase-labeled goat anti-mouse (WB, SeraCare, 074-1806), Peroxidase-labeled rabbit anti-goat (WB, SeraCare, 14-13-06).
Validation	For Goat or Rabbit anti-ATF4 see PMID: 24291486, 29207036 and also was validated in this paper; For Rabbit anti-TRPM3 see PMID: 27030715 and also was validated in this paper. For Rabbit anti-TRPV1 see PMID: 25849380; For Rabbit anti-TRPA1 see RRID: AB_2759384 and the manufacturer's website; For Rabbit anti-Flag see PMID: 31784520; For Rabbit anti-His see PMID: 31911580; For Mouse anti-TfR, Goat anti-KIF3A, Rabbit anti-KIF3B, Mouse anti-KIF17, Rabbit anti-KIF5B, Rabbit anti-KIF5A, Rabbit anti-KIFC2, Mouse anti-beta Tubulin, Mouse or Goat anti-CGRP, Isolectin B4-FITC, Mouse anti-NF200, Mouse or Rabbit anti-NeuN, Rabbit anti-CXCR4, Rabbit anti-CXCR7 see our previous publications PMID: 31446225, 29588412, PMID: 33433144 and 28072604. The validation data for all antibodies can be found in the manufacturer's website.

Eukaryotic cell lines

Policy information about cell lines

Cell line source(s)	The HEK 293T cell line was derived from embryonic kidney of human. The BV-2 cell line was derived from microglial cells of mice. HEK293T cell line and BV-2 cell line were obtained from American Type Culture Collection (ATCC, Manassas, VA, USA).
Authentication	STR analysis were chosen to perform the authentication.
Mycoplasma contamination	All the cell lines did not detect any mycoplasma contamination.
Commonly misidentified lines (See ICLAC register)	N/A

Animals and other organisms

Policy information about studies involving animals; ARRIVE guidelines recommended for reporting animal research

Laboratory animals	C57BL/6 (male; 4-12 weeks) mice were obtained from the Institute of Experimental Animals of Sun Yat-sen University. Atf4+/- and Trpm3-/- mice (male and female; 4-12 weeks) with a C57BL/6 background were purchased from the Cyagen Biosciences Inc.
Wild animals	None of wild animals involved in the study.
Field-collected samples	No field collected samples were used in the study.
Ethics oversight	All animal experimental procedures were approved by the local Animal Care Committee (Research Ethics Committee of Guangdong Provincial People's Hospital, Guangdong Academy of Medical Sciences) and were carried out in accordance with the guidelines of the National Institutes of Health on animal care and ethical guidelines.

Note that full information on the approval of the study protocol must also be provided in the manuscript.
